# 1207. Impact of a switch from ciprofloxacin to ceftriaxone on infectious complications after transrectal ultrasound-guided biopsy of the prostate

**DOI:** 10.1093/ofid/ofad500.1047

**Published:** 2023-11-27

**Authors:** Michelle T Hecker, Curtis Donskey, Rafael Mendo-Lopez, Irma Lengu, Carvell Nguyen

**Affiliations:** The MetroHealth System, Cleveland, Ohio; Cleveland VA Hospital, Cleveland, Ohio; University Hospitals-Case Western Reserve University, Cleveland, Ohio; Metrohealth, Cleveland, Ohio; Metrohealth, Cleveland, Ohio

## Abstract

**Background:**

Transrectal ultrasound-guided biopsy of the prostate (TRUBP), first described in the 1980s, remains a commonly performed procedure. Since approximately 2010, increasing infection rates due to fluoroquinolone-resistant *E. coli* have been reported. These data prompted a change in our recommended antibiotic prophylaxis in March 2014 from ciprofloxacin to ceftriaxone. Limited data are available on whether this strategy leads to improved outcomes or contributes to emergence of infections due to ceftriaxone-resistant organisms.

**Methods:**

We conducted a retrospective, single-center cohort study comparing patients who received ciprofloxacin monotherapy from 1/1/2011 through 2/28/2014 and patients who received ceftriaxone monotherapy from 3/1/2014 through 12/31/2022 as prophylaxis for TRUBP. The primary outcomes were the percentage of procedures with positive blood cultures within 30 days after the procedure and the percentage of urinary tract infections (UTI), defined as a positive urine culture, within 30 days after the procedure. Secondary outcomes included the percentage of procedures with UTI due to specific organisms, including *E. coli*, fluoroquinolone-resistant *E. coli*, and extended-spectrum beta-lactamase producing (ESBL) *E. coli* within 30 days after the procedure.

**Results:**

Of 2,910 total TRUBP procedures, ciprofloxacin was used for prophylaxis in 653 procedures in 578 patients, and ceftriaxone was used for 2,257 procedures in 1,794 patients. As shown in Table 1, patients receiving ceftriaxone prophylaxis had significantly fewer positive blood and urine cultures in the 30 days after the procedure and significant reductions in positive urine cultures with *E. coli*, fluoroquinolone-resistant *E. coli*, and non-*E. coli* Enterobacteriaceae.

**Conclusion:**

Our findings suggest that in the setting of increasing rates of fluoroquinolone resistance in *E. coli*, switching from ciprofloxacin to ceftriaxone for TRUBP prophylaxis may result in fewer infectious complications without increasing the risk for emergence of infections due to ceftriaxone-resistant organisms.

**Disclosures:**

**All Authors**: No reported disclosures

